# Septic cardioembolic stroke secondary to infective endocarditis in a young patient with rheumatic heart disease: a case report

**DOI:** 10.1093/omcr/omae083

**Published:** 2024-08-06

**Authors:** Helena Agostingo Buque, Evangelina Namburete, Deise Catamo Vaz, Frederico João Sebasteão, Yanina Baduro, Elder Lorenzo Rosales, Nachan Arroz, Lazara Bacallao, Damiano Pizzol, Lee Smith

**Affiliations:** Department of Neurology, Maputo Central Hospital, 1653 Avenida Eduardo Mondlane, 1100, Mozambique; Department of Internal Medicine, Maputo Central Hospital, 1653 Avenida Eduardo Mondlane, Maputo, 1100, Mozambique; Department of Neurology, Maputo Central Hospital, 1653 Avenida Eduardo Mondlane, 1100, Mozambique; Department of Neurology, Maputo Central Hospital, 1653 Avenida Eduardo Mondlane, 1100, Mozambique; Department of Neurology, Maputo Central Hospital, 1653 Avenida Eduardo Mondlane, 1100, Mozambique; Department of Neurology, Maputo Central Hospital, 1653 Avenida Eduardo Mondlane, 1100, Mozambique; Department of Neurology, Maputo Central Hospital, 1653 Avenida Eduardo Mondlane, 1100, Mozambique; Department of Neurology, Maputo Central Hospital, 1653 Avenida Eduardo Mondlane, 1100, Mozambique; Operational Research Unit, Doctors with Africa, Beira, Rua Fernao Mendez Pinto 165, Ponta Gea, Beira, 1363, Mozambique; Centre for Health, Performance and Wellbeing, Anglia Ruskin University, Young Street, Cambridge, Cambridgeshire CB1 2LZ, United Kingdom

**Keywords:** infectious diseases and tropical medicine, cardiology and cardiovascular systems

## Abstract

The risk of stroke due to infective endocarditis is particularly high during the first week. Moreover, in low-resource settings where imaging access is limited, and diagnostic pathways are inaccurate the risk further increases. In addition to antibiotic therapy, treatment may include intravenous thrombolysis, with high risk of hemorrhagic complications in patients with infective endocarditis or mechanical thrombectomy. We report here a case of a 24-year-old male with rheumatic heart disease presenting a septic cardioembolic stroke secondary to infective endocarditis that was successfully treated in a low-resource setting.

## Introduction

Cerebral ischemic events are among the most common presenting symptoms and a main cause of death of infective endocarditis, although stroke secondary to infections remain a rare condition [1]. Although the increase access to advanced neuroimaging allows the detection of silent early-stage cerebral embolism, clinically manifest stroke remains the classic presentation especially in low-resource settings where the imaging access is limited and diagnostic pathway often inaccurate [2]. Moreover, it is estimated that the risk of stroke due to infective endocarditis remains high (4.8/1000 patient-days) during the first week of antibiotic therapy and then declines rapidly [3]. Stroke secondary to infections is likely a result of embolism by migration of fragments vegetation and/or mycotic aneurysm rupture [1]. The main cerebrovascular complications include meningitis, intracerebral abscess, encephalopathy, hemorrhage and aneurysms with consequent high risk for intracranial bleeding [4]. In infective endocarditis the higher rate of cerebral embolic events are associated with *Staphylococcus aureus* when the anterior leaflet of the mitral valve is affected [5]. Interestingly, the most common location of ischemic events is the middle cerebral artery although also multifocal or distal ischemic events may occur [5]. Based on clinical and functional presentation and health general status, in addition to antibiotic therapy, treatment may include intravenous thrombolysis or mechanical thrombectomy [5].

We report here a case of a 24-year-old male with rheumatic heart disease presenting a septic cardioembolic stroke secondary to infective endocarditis that was successfully treated in a low-resource setting.

## Case report

**Figure 1:**
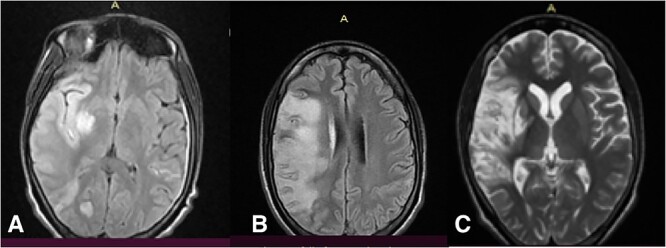
Magnetic resonance showing the cerebral lesion at admission (**A**) and after 14 (**B**) and 33 (**C**) days.

A 24-year-old male presented with sudden onset of muscle strength loss in the left hemi body, found fallen early in the morning, complaining of intense holocranial headache and fever, followed by drowsiness and hypo-responsiveness. He reported that he was attending outpatient follow-ups at the local health center for two months due to epigastric pain diagnosed as gastritis. He was previously healthy, with no smoking or alcohol habits, nor chronic diseases. He was not taking corticosteroids or other immunosuppressive medications. The examination showed drowsiness, clear and coherent speech, with gaze diverted to the right, mydriatic pupils reactive to light, deviation of the lip commissure to the left and facial asymmetry with facial paralysis to the left. Gait not assessable, with muscle strength at 1/5, with neck stiffness and preserved sensitivity. His body temperature was 38.9°C, blood pressure 90/55 mmHg, heart rate 90 bpm. A 5/6 murmur was detected in the 2^nd^ and 5^th^ intercostal space in the left mid-clavicular line, with thrill throughout the precordial area. Biochemistry revealed leukocytosis with neutrophilic predominance, moderate anemia, mild hyponatremia and hypochloremia. Malaria, HIV and Covid-19 tests were negative, but he had a positive blood culture for *S. aureus*, sensitive to Ceftriaxone. Cardiac ultrasound revealed carditis with vegetations on the mitral and aortic valves. Magnetic resonance imaging (MRI) showed an extensive lesion in the area of the right middle cerebral artery and occipital with extensive perilesional edema (Fig. 1A). He was first treated with Ceftiaxone 2 g twice per day, Gentamicin 160 mg/day, Mannitol 20% 80 ml each four hour, lactate ringer 2 l/day, Aspirin 100 mg/day, Simvastantin 20 mg/night, Lisinopril 5 mg/day, Paracetamol 1 g each six hours and Omeprazole 20 mg/day.

After 5 days of treatment, there was a clinical improvement, he was awake, cooperative, apyretic, with no neck stiffness or headache; The focal neurological deficit with left hemiparesis persisted and he started physiotherapy. On the 14^th^ day, he had improvement in muscle strength with 3/5 in the upper limb and 2/5 in the lower limb. Control MRI showed edema reduction with areas of cerebritis. (Fig. 1B). Ultrasound confirmed chronic rheumatic heart disease with severe mitral insufficiency and mild to moderate aortic insufficiency. Ceftriaxone was suspended and ciprofloxacin 500 mg twice per day was started for 14 days. On the 33^rd^ day MRI showed hypersignal in the territory of the middle cerebral artery, with rediffusion and without cerebral edema (Fig. 1C). On the 35^th^ day he was discharged with outpatient follow-up.

## Discussion

The most important factor in managing cardioembolic stroke secondary to infective endocarditis is timing in terms of proper diagnosis and adequate treatment. In particular, the early administration of effective intravenous antibiotics allows to reduce mortality and morbidity from embolic complications and heart failure [5]. In the present case, the patient presented a full-blown symptomatic stroke including sudden and complete hemiparesis associated with fever, neck stiffness, altered level of consciousness and heart murmur. Such presentation raised the suspicion of cardiac involvement and, as expected, diagnosed, with subsequent MRI confirmation, a cardioembolic stroke secondary to infective endocarditis likely developed during previous weeks based on reported clinical history. On the one hand, this reiterates the lack of adequate tools for diagnosis, the lack of trained medical professionals and the absence of an effective referral system in low-resource settings. On the other hand, it significantly reduced the chances of a favorable outcome for the prognosis. In this case, ultrasound played a crucial role in diagnosis orientation, in line with previous evidence on the versatility of this diagnostic tool that can be easily employed in extremely resource-limited settings where radiological and microbiological investigations are scarce [6]. Moreover, the patient presented with large vessel occlusion and, at the time of writing, there is no consensus on the gold standard management for similar cases. Indeed, for in-patients with acute ischemic stroke treatment with intravenous thrombolysis is suggested; in patients with infective endocarditis particular attention must be paid due to the high risk of hemorrhagic complications [7]. An option to be considered for these patients could be a mechanical thrombectomy [7]. While the first option was not performed by choice, there was no possibility to consider the second option due to lack of equipment. In low-resource settings, limited equipment is common, reducing favorable prognosis [8]. Immediately, even before having confirmation of positive culture for *S. aureus*, the patient commenced an intravenous antibiotic therapy with Ceftriaxone and Gentamicin which proved to be lifesaving in this case. Indeed, the patient conditions improved significantly after 4–5 days of therapy institution and no complication such as heart failure or valve rupture developed.

This successful case highlights on one hand the proper management of such complicated and late-stage condition, and on the other hand the necessity to continuously train and support health professionals across low-resource settings. Onsite healthcare workers training is crucial to avoid “brain drain” as well as to avoid training on different standards of practice and ultimately to encourage a sense of belonging and professional growth. In particular, for this specific condition, a recent history of strep infection or rheumatic fever is the key to hypothesize the diagnosis of rheumatic heart disease. Moreover, symptoms of rheumatic fever vary and typically begin one to six weeks after a bout of strep throat and, in some cases, the infection may have been too mild to have been recognized, or it may be gone by the time the person sees a medical practitioner. These considerations reinforce the importance of appropriate training for health professionals especially in remote areas and make mandatory a call to action to all involved institutions to improve and increase the efforts to achieve universal health care coverage.
